# Editorial: Recent advances in endometriosis: from Bench to clinical application

**DOI:** 10.3389/fendo.2025.1570284

**Published:** 2025-02-19

**Authors:** Iveta Yotova, Achmad Kemal Harzif

**Affiliations:** ^1^ Department of Obstetrics and Gynecology, Medical University of Vienna, Vienna, Austria; ^2^ Department of Obstetrics and Gynecology, Faculty of Medicine Universitas Indonesia, Dr. Cipto Mangunkusumo Hospital, Jakarta, Indonesia

**Keywords:** endometriosis, diagnosis, pathogenesis, treatment, disease models

Endometriosis is a chronic, estrogen-dependent disease denoted by endometrial-like growths outside of the uterus, usually on organs in the peritoneal cavity. Endometriosis is often associated with chronic gynecological or pelvic pain, and an increased likelihood of reduced fertility, with reports indicating that up to 10% of reproductive age women are affected by the disease (1). Endometriosis pathogenesis involves complex molecular mechanisms, including genetic, epigenetic, hormonal and immunological dysregulation, oxidative stress and fibrogenesis that remain to be fully deciphered (2). Patients have heterogeneous, non-specific symptoms making definite diagnosis difficult without explorative laparoscopic surgery, although non-invasive imaging techniques, such as magnetic resonance imaging (MRI) and ultrasound, may in some cases facilitate diagnosis. This difficulty in diagnosis can lead to delays in the start of treatment, illustrating the need for improved methods for diagnosis (1). Therefore, studies that hold promise for non-invasive biomarkers for diagnosing endometriosis and/or progression, such as epigenetic regulators, gene profiles or specific proteins could eventually complement imaging in enabling a comprehensive, non-invasive diagnostic approach. Treatment of the disease is largely limited to laparoscopic surgery to remove lesions, and hormonal and pain relief medications (3). Improvements to diagnosis and treatments may be aided by a better understanding of the pathogenesis of the disease, ensuring a high quality of personalized patient care in the future.

In furtherance of this vision, this Research Topic brings together a unique blend of original research, study protocols and review articles, drawing attention to the latest advances in understanding endometriosis pathogenesis, disease-associated comorbidities, and progress made toward non-invasive diagnosis and treatment.

Since their discovery in the early 1980s, exosomes have been indicated to be a universal form of cell-to cell communication, with high potential as therapy delivery systems and biomarkers in wide range of diseases including endometriosis (4). The comprehensive review by Wang et al. provides an overview of the role of exosomal long-noncoding RNAs in the regulation of different aspects of endometriosis pathogenesis, lesion neovascularization and endometriosis-associated infertility, and discusses their potential as non-invasive diagnostic biomarker for the disease.

Together with chronic pain, endometriosis associated infertility is one of the main factors affecting the quality of life of affected women. Possible causes of infertility are molecular changes in endometrium, chronic intraperitoneal inflammation, progesterone resistance, disturbed folliculogenesis, reduced ovarian reserve, dysfunctional uterotubal motility, hormonal and immunological changes affecting embryo implantation, oogenesis and endometrial decidualization. In their comprehensive review article, Fan et al. summarize the current knowledge of the role of impaired granulosa cell function on oocyte quality in women with endometriosis. One of the ovulatory disfunction subtypes considered to be a cause of endometriosis-associated infertility is so-called luteinized unruptured follicle syndrome (LUFs) (5). However, the precise underlying reasons for association of LUFs with endometriosis remain unknown. In their original work, Geng et al. used an endometriosis mouse model to uncover molecular basis of LUFs in endometriosis. Using a range of *in vivo* and *in vitro* experiments they provide evidence that attenuation of LHCGR in granulosa cells is involved in the increased incident of LUFs in endometriosis via mechanisms leading to sustained COX-2 expression. The original work of Pei et al. links impaired endometrial stroma cells decidualization in women with endometriosis with the deregulation of autophagy associated with an increased Hippo/YAP and reduced mTOR signaling. An interesting study by Li et al. reports the negative influence of oviductal obtained vesicles (oEV) from women with endometriosis on early embryo development *in vitro*. The authors show significant changes in blastocyst transcriptome, with a major reduction of oxidative phosphorylation in murine blastocysts co-cultured with oEVs of women with the disease associated with increased cell death. Conceptually innovative, the work of Xiang et al. evaluates the changes in secretory phase eutopic endometrial transcriptome after surgical removal of lesions from women with severe endometriosis. Based on *in silico* modeling, the authors speculate that the postsurgical changes in the endometrial transcriptome may cause functional and immunological changes that improve endometrial receptivity.

The interplay between the gut microbiome and estrogen in the pathogenesis of endometriosis was evaluated by Alghetaa et al. Using a mouse model of endometriosis, the authors showed that significant changes in immune cell response in peritoneal cavity of endometriosis mice are strongly associated with changes in the gut microbiota, with an increase in bacterial species producing less short chain fatty acids metabolites compared to respective controls. These changes were associated with an accelerated metabolic rate in peritoneal inflammatory immune cells, suggesting that modulating the gut microbiota in women with endometriosis might be a powerful therapeutical strategy for treatment of endometriosis associated pain and inflammation. The advantage of in silico modeling of existing expression profiling data, followed by *in vitro* validation to aid understanding of the immune responses in endometriosis is demonstrated by the work of (Lv et al.). Here the authors identified and validated 10 central genes in an endometriosis co-expression network analysis, so-called “hub” genes, which were significantly correlated with specific disease-related immune cell infiltration and/or immune-related pathways. A similar experimental strategy was used by Pei et al. to study the molecular mechanisms of regulation of autophagy in endometrial endometriosis stroma cells. Autophagy plays an essential role for cellular response to stress conditions and ensures tissues repair and survival in health and disease (6). Hypoxia is the main stress factor that endometrial cells face in the peritoneal cavity during menstruation, and therefore, tight regulation of autophagy is an essential mechanism for endometriosis lesion development in women with endometriosis. However, the role of dysregulated autophagy in impaired endometrial receptivity in women with endometriosis is still largely unknown. In their paper, Pei et al. reported the association of Yes-associated protein (YAP) with the regulation of mTOR signaling pathway in eutopic endometrial stroma cells of women with endometriosis, and showed that YAP-mediated suppression of mTOR autophagy signaling leads to improved decidualization of eutopic endometriosis stroma cells.

Hypoxic conditions are known to favor the increase of Reactive Oxygen Species (ROS) and oxidative stress, both recognized as important pro-inflammatory mediators in endometriosis associated neurogenic pain (7). Therefore, antioxidant therapies for effective management of ROS and inflammation are of particular interest for treatment of endometriosis-associated pain and inflammation. In their randomized, triple-blind, placebo-controlled clinical study Rostami et al. tested the therapeutical potential of the strong antioxidant astaxanthin (AST) in women with the disease. The study showed that AST supplementation significantly improves oxidative stress serum markers and reduces the levels of pro-inflammatory cytokines in follicular fluid of women with endometriosis, followed by an increase in the quality and the number of oocytes, leading to improvement of assisted reproductive treatment (ART) outcomes. These findings indicate that AST pretreatment may be a suitable therapy for infertile endometriosis patients undergoing ART.

Since high fat diet (HFD) can induce chronic pain and inflammation (8), in their study the group of Herup-Wheeler et al. asked whether and how unhealthy HFD can influence endometriosis-associated pelvic pain and inflammation. Using a diet-induced obesity mouse model of endometriosis, the authors showed that HFD alone might not establish a local inflammatory environment in the pelvic cavity, but can contribute to existing endometriosis-related chronic inflammation. This leads to the aggravation of endometriosis-associated abdominal hyperalgesia due to significant increase in pro-inflammatory cytokine levels, macrophages in the peritoneal cavity, neuromodulators in the root ganglia, and dysbiosis of gut microbiota of endometriosis HFD mice, compared to obese mice without endometriosis.

Recent experimental evidence suggest that changes in glucose, lipid, amino acid and nucleotide metabolism and their molecular regulators on cellular and systemic levels are closely related to the development of endometriosis and/or with an increased risk for the disease (9). The population- based study of Liu et al. evaluated the association of the triglyceride-glucose (TyG) index with susceptibility to endometriosis in a cohort of 1590 eligible participants and found that a higher TyG index is significantly associated with increased endometriosis risk. This indicates that TyG index can potentially be used as risk assessment molecular biomarker for endometriosis and may serve as a guide to develop future prevention strategies. The studies by Wójtowicz et al. and Zyguta et al. assessed the potential of adipokines as diagnostic markers for endometriosis. These studies indicated that an integrated multi-body fluid approach on large study cohorts is needed for successful identification and validation of clinically applicable non-invasive diagnostic biomarkers for endometriosis.

An intriguing study by the group of Bai et al. sheds a light on the causal relationship between leukocyte telomere length (LTL) and endometriosis. The authors performed Mendelian randomization study and showed that longer LTL are associated with an increased risk of endometriosis. This novel finding opens potential new aspect for investigating the genetic risk factors of the disease.

To conclude, this Research Topic represents a series of papers ranging from basic research to clinical studies that provide a valuable addition to the expanding knowledge of endometriosis.
